# The Effect of Iron Fortification on Iron (Fe) Status and Inflammation: A Randomized Controlled Trial

**DOI:** 10.1371/journal.pone.0167458

**Published:** 2016-12-06

**Authors:** Jingqiu Ma, Qianqian Sun, Jinrong Liu, Yanqi Hu, Shanshan Liu, Jie Zhang, Xiaoyang Sheng, K. Michael Hambidge

**Affiliations:** 1 Department of Children and Adolescents Health Care, Xin Hua Hospital, Shanghai Jiao Tong University School of Medicine, Shanghai Institute for Pediatric Research, MOE-Shanghai Key Laboratory of Children’s Environmental Health, Kongjiang Road, Yangpu District, Shanghai, China; 2 University of Colorado, Denver, Colorado, United States of America; Universidade de Sao Paulo, BRAZIL

## Abstract

**Background:**

Iron deficiency (ID) is common in toddlers in developing countries. Iron fortified or meat-based complementary foods may be effective to prevent ID.

**Objective:**

Our objective was to compare iron status at 18 months and growth from 6 to 18 months in rural poor toddlers fed 3 different complementary foods.

**Methods:**

The study was nested within a larger trial in which 6-month-old infants were randomized to receive 50g/d meat (MG), an equi-caloric fortified cereal supplement (FG) or local cereal supplement (LG) for 1 year. Hb, sTfR, HsCRP, ferritin and AGP were measured in 410 blood samples collected by a random sampling (MG, 137; FG, 140; LG, 133); calprotectin was measured in feces. Body iron = -[log (sTfR ×1000/ferritin)-2.8229] /0.1207. ID = ferritin<12ug/L.

**Results:**

The toddlers in FG had the significantly highest levels in serum ferritin and body iron (*P* = 0.043, 0.004), and the rates of both ID and iron deficiency anemia (IDA) were the lowest in FG (*P* = 0.010, 0.021). The rate of systemic inflammation in FG was 30.71%, which was the highest among three groups (*P* = 0.042). No intervention effects on either the rates of ID and IDA or iron stores (serum ferritin and body iron) were shown in MG. The change in length-for-age z scores (LAZ) from 6 to 18 months among three groups was significantly different (*P* = 0.021) and a smaller decrease of LAZ in MG and a larger decrease of LAZ in FG were observed.

**Conclusion:**

Iron fortified cereal improved iron status of poor rural toddlers but was also associated with systemic inflammation which was likely to impair their growth.

## Introduction

Infants and young children have a high risk for developing iron deficiency (ID), because they have high demand for iron during the period of rapid growth. However, ID delays or impairs children’s mental and physical development, potentially leading to long-term adverse effect on human health. In China, the prevalence of ID was estimated to be 40% in children aged between 7 months and 7 years old based on a large epidemiologic investigation carried out from 2000 to 2001[1]. There have been many efforts to fight ID and anemia over the past two decades, but in spite of these efforts the conditions are still common [2].

ID tends to be aggravated by the insufficient intake of iron from daily diet [3]. The concentration of iron in human milk is relatively independent of maternal intake, therefore, older infants are mostly dependent on complementary foods to meet iron intake requirements [4]. However, traditional feeding practices, including reliance on cereals and plant-based diets, cannot complement the requirement gap for iron in human milk [5].

Iron fortification food introduced to infants as a daily complementary food can be an effective strategy to increase iron stores and prevent ID [6–8]. However, a recent study reported that iron fortification was ineffective to improve iron status and anemia, while it is shown to be associated with increased gut inflammation [9].

Alternative to the plant-based complementary foods, meat is an excellent source of bioavailable iron, which may improve iron shortage in the diets of older infants and young children. However, randomized trials examining the effects of meat intake on iron status in infants are few [6], especially those initiated in 6-month-old infants [10, 11].

In this study, our objectives were to determine 1) the efficacy of complementary foods including iron-fortified cereal and meat, in improvement of iron status; 2) the effect of different complementary food choices on intestinal and systemic inflammation in a poor rural area setting. Our hypothesis was that the consumption of iron-fortified cereal or intake of meat would improve the biochemical iron status of healthy infants and young children.

## Subjects and Methods

### Study design

The study was a cross-sectional sub-sample nested within a larger intervention trial in which the effect of meat was evaluated as the primary complementary food on linear growth between 6–18 months of age compared with equi-caloric quantities of rice cereal or of micronutrient-fortified rice cereal as controls [12].

This cluster-randomized, non-masked, controlled efficacy trial (S1 File) was conducted from March 2009 to December 2011 in a poor rural area, Xichou County located in Yunnan province of China [12]. In the study region, various types of plant-based gruels made from cereal grains or starchy roots and tubers are common early complementary foods offered to infants. Xichou County has an average elevation of 1400 meters above sea level. The study included 60 villages (clustered) in 9 districts in Xichou County.

The specially trained community doctors in each of the small communities within each village were in charge of the intervention to participants. These doctors were also responsible for assessing compliance and morbidity. They reported to and collected fresh supply of intervention foods each week from the village hospitals, where weekly supplies of fresh frozen pork and cereal supplements from the Xichou Maternal and Child Health Service were distributed. The participants were seen by the assessment team at the nearest hospital at baseline and at 3-month intervals. In addition to anthropometric data, morbidity and dietary data were reported. The larger trial in which this study was nested was registered at clinical trials.gov as NCT00726102.

Eligibility criteria for infants included healthy singleton infants between 3–5 months of age, born between 37 to 42 weeks gestational age, with birth weight >2 000 g, with no metabolic or physical problems, lack of acute or chronic illness, and being exclusively breastfed.

### Interventions

The intervention meat of choice was selected as fresh pork in this population. Fresh certified-safe lean pork was purchased weekly, minced and accurately weighed into daily 50g aliquots containing 1 mg Fe in individual plastic bags, which were stored frozen until transported weekly to the district hospitals serving the meat villages. The community doctors serving the meat villages collected the required number of 50g aliquots of meat weekly from the district hospital. The meat was stored frozen by the community doctors in their own facilities and 50g servings were distributed every 2^nd^ day to the participants’ homes. Meat was cooked by parents and fed to the infant. Participants in the meat group (MG) were encouraged to consume 50g meat (calories: 80 kcal) each day. If the days’ supply of cooked meat was not completely consumed in one serving, the mothers kept the remainder for a maximum of 2 h and fed again to their infants.

The quantity of cereal including fortified or local cereal is designed to be equi-caloric to the daily supply of pork. Commercial infant rice cereals (Nestle, fortified with iron, zinc and vitamin B_12_) were bought. The chemical form of Fe and Zn in fortified cereal was ferrous fumarate and zinc sulfate. Participants in fortified cereal group (FG) were encouraged to consume 20g fortified rice cereal (calories: 80 kcal) each day. The iron content in the fortified rice cereal of 20g was 1.10 mg. Local rice cereal was made from a mixture of glutinous rice flour, white granulated sugar and honey. Participants in local cereal group (LG) were encouraged to consume 20g local rice cereal (calories: 80 kcal) each day. The iron content in the local rice cereal of 20g was 0.04mg. Supplies of these control foods were provided weekly to the six hospitals serving all participating villages, from where they were also collected and distributed by the participating community doctors weekly.

Consumption data for any of the intervention were checked and recorded by the community doctors at each visit, i.e. every 2^nd^ day for the meat participants and weekly for the cereal participants. Operation manual of the study is shown in S2 File (Third Version Nutrition Monitoring of Young Children).

### Participants

**Fig 1:**
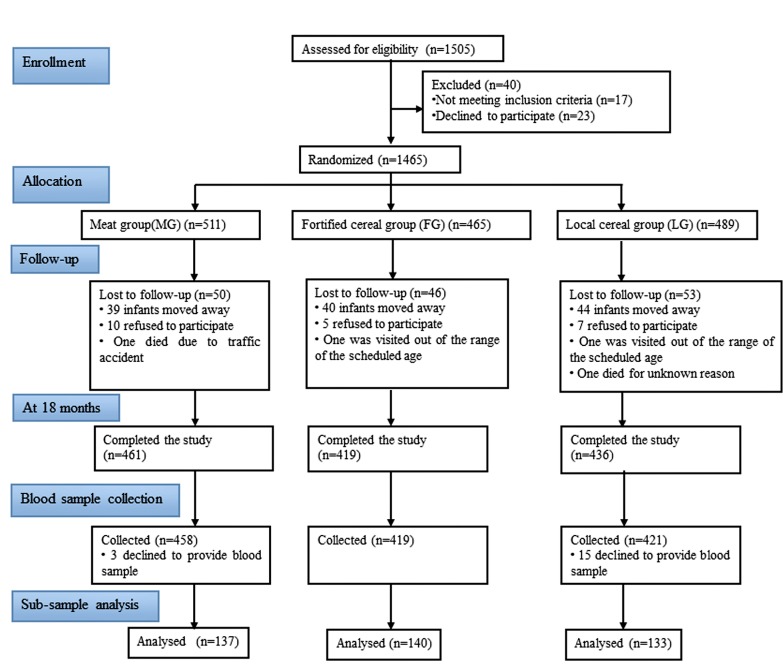
Participant flow through the study.

The flow of study subjects is shown in Fig 1. A total of 1488 infants met the eligibility criteria and 1465 infants entered the study at baseline (6 months), including 511 in the MG, 465 in FG and 489 in LG. During the intervention period from 6 months to 18 months, 50 infants in MG left the study, of whom 39 infants moved away, 10 refused to participate and one died due to traffic accident; 46 infants in FG left the study, of whom 40 infants moved away, 5 refused to participate, and one was visited out of the range of the scheduled age; 53 infants in LG left the study, of whom 44 infants moved away, 7 refused to participate, one was visited out of the range of the scheduled age and one died for unknown reason. As a result, a total of 1316 children completed the study at 18 months, including 461 in MG, 419 in FG and 436 in LG.

Three children in MG and 15 children in LG declined to provide blood samples. At last, a total of 1298 children aged 18 months provided blood samples, including 458 in MG, 419 in FG and 421 in LG, without infection of the upper respiratory tract, diarrhea or other acute infection.

### Blood and stool sample collection

At 18 months, peripheral venous blood samples of 5-mL were collected by trained and experienced phlebotomists into a trace element-free, additive-free evacuated tube. Serum was divided into two aliquots and then stored at -20°C immediately, transported frozen, at last stored at -80°C until analysis in Shanghai Key Laboratory of Children’s Environmental Health. The fresh morning stool samples were also collected at 18 months for the detection of fecal calprotectin and stored at -20°C.

### Sub-sample size

The sub-sample sizes were calculated on the basis of the prevalence of iron deficiency which was estimated to be 40% in children aged between 7-month and 7-year-old in China based on the data from a large epidemiologic investigation done from 2000 to 2001[1]. To detect a clinically important reduction in the prevalence of iron deficiency from 40% to 20% between the local cereal group and the fortified cereal group, blood samples of 133 children were needed in each group to achieve a power of 90% at a 5% level of significance, which allowed for 20% attrition. A simple random blood sample was taken from MG, FG and LG by two investigators who were not involved in the recruitment and data collection. Randomization was achieved by the use of a computer-generated random assignment process.

### Biochemical assessment

Hemoglobin and mean corpuscular volume (MCV) were measured in whole blood with an automated hematology Analyzer on the day of blood sampling. Serum ferritin, serum soluble transferrin receptor (sTfR), highly sensitive C-reactive protein (Hs-CRP) and α_1_-acid glycoprotein (AGP) concentrations were measured at the Department of Clinical Laboratory, Xinhua Hospital, Shanghai Jiao Tong University School of Medicine. The individuals who carried out the laboratory analyses were unaware of the participants’ group assignments.

Serum ferritin, sTfR and AGP concentrations were detected by latex-enhanced immunoturbidimetric assay on a BN II system (Siemens Healthcare Diagnostics, Germany). An Hs-CRP was measured on a Hitachi 7600-120E automated biochemistry analyzer by immunoturbidimetric assay (Orion diagnostica, Finland).

Fecal calprotectin was detected by enzyme-linked immunosorbent assay (ELISA) test (Bühlmann Laboratories AG, Basel, Switzerland) and expressed as micrograms per gram (dried stool).

ID was defined as serum ferritin < 12ug/L [2, 13, 14]. Iron deficiency anemia (IDA) was defined as serum ferritin < 12ug/L and hemoglobin < 115g/L (Xichou county in Yunnan province has an average elevation of 1400 meters above sea level, so the lower limit of normal was adjusted to 115g/L [2, 15]). Body iron [6] was calculated as follows: body iron (mg/kg) = –[log (serum transferrin receptor in mg/L ×1000/serum ferritin concentration in ug/L)-2.8229]/0.1207. Systemic inflammation was defined as either an AGP concentration > 1.2g/L or an Hs-CRP concentration > 3mg/L [9] and was used to adjust for the influence of inflammation on serum ferritin [16].

### Anthropometric variables

All anthropometric measurements including length, weight, and head circumference at the age of 6, 12 and 18 months were measured using standardized procedures by an assessment team. Infants were weighed using an electronic pediatric scale to the nearest 0.005 kg with light clothing and without shoes; length was measured using a pediatric length board to the nearest 0.1cm with the toddler in a recumbent position; head circumference was measured by Seca measuring tape to the nearest 0.1cm. Standardization exercises for inter- and intra-measurer reliability in weight, length and head circumference were performed in which each toddler was measured twice. If the differences between two measurements in weight more than 10 g, in recumbent length more than 0.4 cm and in head circumference more than 0.2 cm, a third measurement must be taken. The first or last record was reported.

Weight-for-age z scores (WAZ), length-for-age z scores (LAZ), weight-for-length z scores (WLZ) were calculated according to the 2006 WHO Child Growth Standards using WHO Anthro 2009 software [17]. Underweight, stunting, wasting were defined as WAZ<-2, LAZ<-2, and WLZ<-2, respectively.

### Ethical considerations

Written informed consent was obtained from the parents and main caregivers on behalf of the infants enrolled in this study. The study protocol was approved by the Ethics Committee of Xinhua Hospital, affiliated to Shanghai Jiao Tong University School of Medicine (No. 08–0768) and conducted in accordance with the revised Declaration of Helsinki.

### Statistical analysis

The biochemical iron indexes and inflammatory markers were analyzed using descriptive and frequency statistics in SPSS 13.0 for windows. Descriptive statistics are presented as mean±SD for normally distributed continuous variables or median (interquartile range) for data not normally distributed. Log transformations were used where residuals were skewed or exhibited nonconstant variance. Equal variance was checked using Levene’s test. One-way ANCOVA was used to test the effect of group on primary biochemical iron indexes and inflammatory markers (after log transformations for serum ferritin, Hs-CRP, α1-Acid glycoprotein, transferrin receptor, and fecal calprotectin) and age was controlled as a covariate. Other continuous variables including LAZ, WAZ and WLZ at 6 and 18 months were also tested using One-way ANCOVA and sex, birth weight of toddlers, mother’s pre-pregnancy BMI were adjusted as confounders. LSD multiple comparison was used for post hoc analysis if the model was significant. Chi-square tests were used for categorical variables. Serum Hs-CRP and α_1_-Acid glycoprotein were used to remove the influence of inflammation from biochemical iron indexes.

All statistical tests were two-tailed and *P* values <0.05 were considered statistically significant. Anthropometric and hematologic results are reported only for the subsample.

## Results

### Anthropometric outcomes of sub-sample

410 blood samples were taken by a simple random sampling from 1298 blood samples stored at -80°C in Shanghai Key Laboratory of Children’s Environmental Health, 137 in MG, 140 in FG and 133 in LG, respectively. The mean ages of toddlers among three groups were (17.87±0.1), (17.95±0.2) and (17.93±0.2) months, respectively. There was significant difference of mean age among three groups (*P* = 0.001). The percentages of boy among three groups were 55.47%, 49.29% and 51.13%, respectively, and had no significant difference (*P* = 0.572). (Table 1) (S3 File)

**Table 1:** Comparison of anthropometric outcomes by intervention group.

Variables	Meat group (n = 137)	Fortified cereal group (n = 140)	Local cereal group (n = 133)	P value
Age (mo)^1^	17.87±0.11	17.95±0.17	17.93±0.18	**0.001**
Male [n (%)]	76(55.47)	69(49.29)	68(51.13)	0.572
WHO Z scores at 6 months^4^				
LAZ	-1.06±0.99	-1.13±0.93	-1.05±0.96	0.456
WAZ	-0.47±1.02	-0.61±0.99	-0.56±1.06	0.601
WLZ	0.34±1.04	0.21±0.95	0.21±0.98	0.677
WHO Z scores at 18 months^4^				
LAZ	-1.47±0.99^2^	-1.83±0.95	-1.67±0.97	**0.024**
WAZ	-0.89±0.93	-1.04±0.89	-0.91±0.88	0.496
WLZ	-0.27±0.92	-0.24±0.84	-0.15±0.82	0.507
Changes in WHO Z scores from 6 to 18 months^4^				
ΔLAZ	-0.41±0.78^2^^,^^3^	-0.71±0.64	-0.62±0.75	**0.021**
ΔWAZ	-0.42±0.61	-0.43±0.58	-0.34±0.73	0.872
ΔWLZ	-0.61±0.90	-0.44±0.78	-0.36±0.86	0.114

1 Mean±SD (all such values).

2 Compared with fortified cereal group, P<0.05

3 Compared with local cereal group, P<0.05

4 One-way ANCOVA used to test difference by an adjustment in sex, birth weight, mothers’ pre-pregnancy BMI.

There was no significant difference in LAZ, WAZ and WLZ at baseline (6 months) among three groups (all *P*>0.05). However, the difference in LAZ at the age of 18 months among three groups was significant (*P* = 0.024) with a highest LAZ was observed in MG after adjusting for sex and birth weight of toddlers, and mother’s pre-pregnancy BMI. Through LSD multiple comparison, it is suggested that LAZ at 18 months in MG was significantly higher than that in FG while no difference was significant either between FG and LG, or between MG and LG. No significant difference in WAZ and WLZ at 18 months existed among three groups (*P*>0.05). (Table 1)

Furthermore, the difference in change of LAZ from 6 to 18 months among three groups was significant (*P* = 0.021) and a smaller decrease of LAZ in MG and a larger decrease of LAZ in FG were observed. The decreases in WAZ and in WLZ from 6 to 18 months among three groups were not significant (*P*>0.05). (Table 1)

### Iron indexes outcomes

The difference in serum ferritin concentration was significant among three groups (*P* = 0.043) with a higher serum ferritin concentration in FG. Through LSD multiple comparison, it is suggested that the serum ferritin concentration in FG was significantly higher than that in LG; no significant difference in serum ferritin concentration was found between either MG and LG, or FG and MG. (Table 2) (S3 File)

**Table 2:** Iron indexes and inflammatory markers in three study groups (18 months).

Variables	Meat group (n = 137)	Fortified cereal group (n = 140)	Local cereal group (n = 133)	P value
Hemoglobin (g/L)^1^	122.3±11.4	121.6±11.7	119.5±12.1	0.150
sTfR (mg/L)^2^	1.61(1.38–1.97)	1.59(1.35–1.83)	1.69(1.38–1.93)	0.783
Hs-CRP (mg/L)^2^	0.45(0.12–1.55)	0.49(0.15–2.88)	0.39(0.07–1.56)	0.578
α_1_-Acid glycoprotein, g/L, (n) ^2^	0.67(0.50–0.90)(83)	0.81(0.59–1.07)(107)	0.65(0.47–1.07)(90)	0.105
Systemic inflammation [n (%)]^3^	25 (18.25)	43 (30.71)	29 (21.80)	**0.042**
Fecal calprotectin, ug/g, (n) ^2^	143.6(51.3–254.9)(37)	232.0(54.07–462.07)(40)	121.8(48.7–359.7)(32)	0.385
Serum ferritin (μg/L)^2^^,^^4^	17.70(10.05–26.35)	18.95(12.30–31.55)^5^	15.20(8.96–26.75)	**0.043**
Body iron (mg/kg)^1^^,^^4^	6.52±3.11	7.68±2.38^5^^,^^6^	6.21±3.46	**0.004**
Iron deficiency [n (%)]	42 (30.66)	32 (22.86)	53 (39.85)	**0.010**
Iron deficiency anemia [n (%)]	11(8.03)	10(7.14)	22(16.54)	**0.021**

1 Mean±SD (all such values).

2 median; interquartile range in parentheses (all such values).

3 Hs-CRP>3mg/L or α1-Acid glycoprotein concentrations>1.2g/L.

4 Excluded subjects with Hs-CRP>3mg/L and α1-Acid glycoprotein concentrations>1.2g/L; for meat, fortified cereal and local cereal groups, respectively: n = 112, 97, and 104.

5 Compared with local cereal group, P<0.05

6 Compared with meat group, P<0.05

There was significant difference in body iron among three groups (*P* = 0.004) and the level of body iron in FG was higher. Through LSD multiple comparison, the body iron in FG was observed significantly higher than that in LG and in MG; however, there was no significant difference in body iron between MG and LG. (Table 2)

There was no significant difference in hemoglobin and sTfR concentrations among three groups. (Table 2)

The percentage of ID in MG, FG and LG were 30.66%, 22.86% and 39.85%, respectively. The differences in prevalence of ID among three groups were significant (*P* = 0.010) with the highest rate of ID in LG, followed by MG and FG. Meanwhile, the percentage of IDA in MG, FG and LG were 8.03%, 7.14% and 16.54% respectively, and the children in LG had the highest rate of IDA (*P* = 0.021). (Table 2)

### Systemic and gut inflammation

In 410 blood samples, there were 75 children with an Hs-CRP concentration > 3mg/L and 41 children with an AGP concentration > 1.2g/L. The rates of systemic inflammation determined by either Hs-CRP> 3mg/L or α1-Acid glycoprotein concentration > 1.2g/L were 23.66% (97/410) in all sub-samples, 18.25% in MG, 30.71% in FG and 21.80% in LG, respectively. Toddlers in FG had the highest rate of systemic inflammation among three groups (*P* = 0.042). However, the concentrations of Hs-CRP and α1-Acid glycoprotein were similar among three groups (*P*>0.05).

A convenience sampling of stool samples from MG (n = 37), FG (n = 40) and LG (n = 32) was used for the detection of fecal calprotectin concentration, and there was no significant difference in fecal calprotectin concentration among three groups (*P*>0.05). (Table 2)

## Discussion

Concern should be raised that the rice-based complementary feeding diet was too low in iron to meet physiological requirements in infants and young children. In the sub-sample, 39.85% of ID toddlers in local cereal group indicated the populations in a poor rural area, Xichou County of China, were at a high risk of iron deficiency. Furthermore, we found the rates of ID and IDA in toddlers of fortified cereal group were 17% lower and 9.4% lower than those in local cereal group. It is suggested that iron fortified cereal as a complementary food between 6–18 months of age is helpful for preventing from iron deficiency in a critical time for brain and mental development, which may have long-lasting adverse effects on behavior, learning, and mental performance. A large-scale randomized effectiveness trial implemented for 12 months in Mexican children concluded that iron-fortified milk was effective at reducing the rates of anemia and iron deficiency [7].

Several studies have reported that toddlers who consume iron-fortified formula show improved serum ferritin concentrations when compared with those who drink non-fortified cow milk [6, 7, 18]. Likewise, our results showed increased serum ferritin concentrations in fortified cereal group. Moreover, we also observed that the body iron level was increased by consumption of iron-fortified cereal, which was similar with the result of a randomized controlled trial [6]. Therefore, iron-fortified cereal is likely an effective strategy to improve the iron stores in toddlers.

A previous study reported even 1 mg of inorganic iron per day given in a wheat product increased the amount of hemoglobin in young children [19]. However, in our study no intervention effect was observed on hemoglobin levels, which was similar with two other studies [6, 18]. In contrast to the high rate of iron deficiency, the relatively lower rate of iron deficiency anemia in this population may contribute to the result. More supplemental iron was stored in the body due to iron depletion rather than be used for hemoglobin synthesis.

As is well known, serum ferritin concentrations are influenced by inflammation. Generally, CRP concentrations were measured to adjust for the influence of inflammation on serum ferritin [6, 18]. A recent meta-analysis supported that measures of both AGP and CRP were needed to estimate the full effect of inflammation and could be used to correct ferritin concentrations [16]. Therefore, both Hs-CRP and AGP concentrations were tested in our study and further AGP concentration>1.2g/L or Hs-CRP concentration>3mg/L was considered as systemic inflammation referring to a recent study [9].

In poor areas, infants and young children are easily accessible to the pathogens especially enteric pathogens that were attributable to unhygienic living conditions. Although there was no clinically evident illness in the current study population in Xichou County, the rate of systemic inflammation in the sub-samples was 23.66%, as high as the rate in a population of sub-Saharan Africa [9].

Furthermore, it is worth noting that the toddlers in FG had the higher percentage of systemic inflammation. The iron fortificant in cereal was non-heme iron with low absorption. The unabsorbed iron fortificant passed into the colon and was potentially available for the gut microbiota. Abundant lactobacilli and other commensal bacteria in the colon provide an important barrier effect against colonization and invasion by pathogens [20]. However, Iron fortification favored the growth of enterobacteria over lactobacilli due to their differing in iron requirements, and the protective barrier may have been weakened [9]. A previous study found that the unbalance of gut microbiota induced by iron fortification was not associated with clear clinical diseases while increased the concentrations of fecal calprotectin, a more specific and sensitive marker for gut inflammation than other systemic inflammatory markers [9]. In our sub-sample, the concentration of fecal calprotectin was measured only in 107 stool samples because of budgetary restrictions, and the result that no significant difference was found in levels of fecal calprotectin among three groups was likely to be associated with the small sample size. Nevertheless, the increased rate of systemic inflammation in toddlers of fortified cereal group indicated the potential adverse effect of iron fortification.

Increased production of pro-inflammatory cytokines is often seen in children with systemic inflammation. Pro-inflammatory cytokines may act individually or in combination to influence child growth through systemic effects and/or a local effect at the level of the growth plate of long bones [21, 22].

Moreover, it had been recognized by several studies implemented in poor areas that the subclinical, asymptomatic infections resulted in stunting in children independent of nutrition factor [23, 24]. In another convenience sub-sample of the study population in Xichou County, the significant negative regression between LAZ at 6 months and fecal calprotectin levels suggested that gut inflammatory factors had a role in growth faltering [25]. As a result, a larger decrease of LAZ between 6–18 months of age observed in fortified cereal group indicated that asymptomatic systemic inflammation induced by iron fortification through favoring the growth of enteric pathogens may have impaired the growth of children.

Meat contains heme iron that is well absorbed and, unlike nonheme iron, its absorption is little affected by dietary factors. However, a randomized control efficacy trial found 2 oz beef supplementation per day was an ineffective strategy for improving the suboptimal iron status in 12-20-mo-old children, but slightly increased the serum ferritin concentration [6]. In our study, pork was used in the meat group, as pork is the most widely used and affordable to every household in China. Nevertheless, pork only contained half as much iron as that in equivalent beef. Similarly, our data indicated that meat intervention did not appear to have an effect on either the rates of ID and IDA or iron stores (serum ferritin and body iron) in a post hoc analysis.

Meat is an excellent resource of nutrients including fats, proteins, vitamin B_12_ and zinc besides iron which were beneficial to child growth [5]. In the sub-sample, a smaller decrease of LAZ from 6 to 18 months in meat group was observed. About 11 grams of protein provided by 50 grams of lean pork may have positive effect on the growth of children. Therefore, though the benefit of supplementing the plant-based complementary foods with meat was insufficient to improve the iron status in this poor rural population, consuming meat-based complementary foods may be effective to reduce the decrease of LAZ.

### Limitations

There are some limitations in the present study. First, the iron indexes and inflammatory markers were not detected at baseline considering ethical issues, so we did not obtain the data of the changes in the above indexes from 6 to 18 months. Second, the comparison result of fecal calprotectin concentration among three groups was somewhat limited by the small sample size and the method of convenience sampling. Third, the study is a sub-sample nested within a cluster randomized trial. Higher sampling error may exist. Last, there may be some unmeasured confounders which probably had some influence on the results.

### Conclusion

Although iron-fortified cereal strategy is likely to improve the iron status in toddlers, it should be implemented with caution in poor and unhygienic areas with high-risk exposure to pathogens because asymptomatic systemic inflammation induced by iron fortification through favoring the growth of enteric pathogens may have adverse effects on the growth of children. There is no evidence that the meat intervention is sufficient to improve the iron status in this poor rural population, but consuming meat-based complementary foods may be effective to reduce the decrease of LAZ.

## Supporting Information

S1 File CONSORT 2010 Checklist. (DOC)

S2 File Third Version Nutrition Monitoring of Young Children. (PDF)

S3 File Iron. (SAV)
