# Supplementary material for: The Effect of Iron Fortification on Iron (Fe) Status and Inflammation: A Randomized Controlled Trial
Source: PLoS One. 2016 Dec 6;11(12):e0167458. doi: 10.1371/journal.pone.0167458 (PMC5140064; doi:10.1371/journal.pone.0167458)
Supplement: S2 File — (PDF) [file pone.0167458.s002.pdf]

Nutrition Monitoring of Young Children in Xi-Chou
Manual of Operations

Section 1: Study Goals, Objectives and Background

1. Study goals:

The goal of this study is to determine the efficacy of a daily intake of locally available, low cost meat as a complementary food for young children aged 6-18 months who are otherwise dependent on continued breast feeding and locally available non-fortified plant foods only. The efficacy will be determined on:

- Linear growth and body weight;
- Incidence/prevalence of infectious disease morbidity;
- Brain growth and cognitive development;
- The quantity of zinc absorbed each day (total absorbed zinc, TAZ);
- Iron, zinc and vitamin B_{12} status (including incidence of both iron deficiency and macrocytic anemia);
- Quantify food and nutrient intake, with special emphasis on the test food;
- Monitor indices of small gut function and chronic immunostimulation.

2. Hypothesis to be tested:

The hypothesis to be tested is that meat as a first and regular complementary food in rural communities lacking access to micronutrient fortified formulas/foods is associated with enhanced linear growth and cognitive development and decreased infectious disease incidence/prevalence and with improved zinc absorption and zinc, iron and vitamin B_{12} status. The primary outcome measure is linear growth, as a strongly-validated index of the burden of disease in children aged less than 5 years.

3. Study design:

The study is an unmasked randomized controlled trial with cluster design in which 60 rural village communities, each with 30 infants, within the County of Xi-Chou, Wen-Shan, Yunnan Province, China will be randomly selected and randomly assigned to meat intervention, micronutrient fortified intervention or control clusters. The meat intervention will be meat, which will be fed under the supervision daily from age 6-18 mo, increasing as accepted to a maximum of 50g/d. Participants in micronutrient fortified intervention and control clusters will receive an equi-caloric plant-based meal (Zn and Fe fortified cereal/legume or rice) under the same community arrangements. Recruitment will be at age 3 mo. Tight control, supervision and

effective separation of test and control clusters will be assured by location of clusters in separate administrative villages and supervision of intervention/control feeds 7 days each week in the community doctors' homes for each administrative village. Infectious disease morbidity will be monitored by community doctors daily (including presence of diarrhea, monitoring of fever when febrile illness appears clinically possible and respiratory rate when participant has cough). Anthropometry and quantitative 24-hr diet will be performed at ages 6, 7,9, 12, 15 and 18 mo; Bayley Scales of Infant Development, III (BSID) will be taken at 12 and 18 mo; biomarker and ancillary assays will be performed in Xi-Chou Women and Children's Hospital (CBCs) or in Shanghai Jiao Tong University from samples obtained at 18 mo. Meanwhile, studies of zinc absorption and gut function tests will be performed in a sub-group of 40 subjects each from meat intervention, micronutrient fortified intervention or control clusters, total 120 subjects. Training and supervision of these assessment teams will be the responsibility of collaborative faculty at Jiao Tong University, working closely with the staff at Xi-Chou hospital. Data will be collected using paper/pencil forms and transferred daily to Xi-Chou Women and Children's Hospital after entering into local data management system; data will be transferred weekly to the data manager at Shanghai Jiao Tong University. The DSMB and the Colorado research team will receive 3-monthly reports. Data will be analyzed by the project statistician at UCDHSC in Denver using SAS software (SAS Institute Inc., Cary, NC) and outcome-specific methods.

4. Study rationale:

Undernutrition during the first two years of life is recognized as a major preventable cause of mortality in children aged one mo to 5 y. In contrast to the intensity of efforts to promote exclusive breast feeding until age 6 mo, optimization of complementary feeding (CF) starting at age 6 mo is only recently receiving equivalent attention with, for example, publication of WHO guidelines. Though these guidelines include feeding of animal source foods (ASF) at least by age 9 mo, the great majority of poor children in developing countries receive little or no ASF in their CF. Except in times of emergency food shortages, adequate macronutrients are typically available from plant foods, provided caregivers have adequate knowledge of how to feed their babies. Recent attention has, therefore, focused on micronutrient deficiencies which are widely considered major contributory factors to the rapid onset of stunting later in the first and in the second years of life, with its associated increased risk of

impaired neurocognitive development, infectious disease morbidity and mortality. Results of recent randomized placebo controlled trials of zinc supplements have provided persuasive evidence of the public health importance of zinc deficiency in young children as a major cause of stunting and of infectious disease morbidity/mortality.

Treatment of putative zinc deficiency with supplements, however, presents major logistical problems as evidenced by the challenges and costs of step-up campaigns to enact the WHO recommendation for zinc supplementation in the management of acute diarrhea. Supplements may also have adverse effects as evidenced by the effects of iron supplements in non-anemic young children in falciparum malarial areas. Sprinkles, with delayed release of micronutrients, have some advantage, but this has been confirmed only for iron. Fortification of food staples is increasingly promoted, but it is difficult to achieve the optimal dose of fortification for all segments of any population. Moreover, there have been some unexpected failures of fortified foods to achieve the hypothesized effects. Perhaps the most compelling reasons for optimizing nutrient intakes from non-fortified, affordable, locally grown foods is either lack of access or very uncertain access to fortified foods or supplements. This emphasizes the dependence on locally grown foods which require major attention to food diversity, especially the inclusion of ASF and specifically meat in order to meet requirements for key micronutrients, notably zinc and iron. Though the benefits of ASF, including meat, in complementary feeding have been recognized, this recognition has been insufficient to inspire programmatic action at any national or global level.

Two ounces (50g) of lean pork a principal source of meat in China where this study will be located, provides 2.5 mg bioavailable zinc. Minced meat mixed with a little polished rice, which has a negligible phytate content, has favorable bioavailability (approximately 0.4 fractional absorption) and the lean pork alone will more than meet physiologic requirements for zinc in young children. Additional zinc will continue to be absorbed from breast milk for most of the duration of this project as well as small quantities from other complementary foods. The quantity of iron provided by 2 oz (50g) lean pork is 1.0 mg. Though the latter is modest compared with the Estimated Average Requirement (EAR), especially that between 6-11 mo of age, the EAR is based on absorption of inorganic iron. No estimates of dietary requirements have been published based on a diet providing mainly heme iron. Absorption of heme iron is very favorable and it is hypothesized that the iron derived

from 2 oz (50g) lean pork will significantly improve parameters of iron status. At least one WHO expert considers it likely that meat will be found to be more efficacious than micronutrient-fortified plant foods (R Bahl, MD, PhD, personal communication).

However, investigations of the efficacy of meat as a first and regular complementary food, a likely scenario in hunter-gatherer cultures have been extraordinarily limited, partly, no doubt, because of the assumed high cost of production in terms of global resources. These assumptions and calculations do not appear to account for the ability of foraging, scavenging animals to survive and grow on waste around human habitations, especially those of the rural poor, making availability of affordable meat a realistic goal. In China, pork is a principal animal meat consumed and pigs can scavenge/forage in rural communities at a cost of $0.30/lb lean meat. If the remaining parts of the animal are sold, this reduces the cost of production of lean meat to approximately $0.15/lb or $0.02/2 oz daily serving.

Alternative meat from local foraging/scavenging birds/animals, e.g. poultry, rabbits, and goats, can be substituted elsewhere on a global basis if efficacy is demonstrated for lean pork in this project. In terms of DALYS and of Net Present Value it is hypothesized that regular intake of meat as a complementary food will compare favorably with alternative strategies. Moreover, this approach offers potential for microfinance initiatives thus empowering women in rural communities, an approach taken by the ENAM project in Ghana. Recent studies that encouraged greater use of meats in young children include especially those of Neumann and colleagues in Kenya and the successful complementary feeding education study in Peru with enhanced growth associated with messages that included the regular use of animal source foods, including liver.

5. Study Timeline:

- 2009.3~2009.12: Recruitment/enrollment of participants (3-5.5 months of age);
- 2009.5~2011.6: Begin intervention (6 months of age), follow-up visits (6-18 months of age);
- 2011.6~2011.12: Data analyses and reporting.

Section 2: Research Ethics

1. Ethics

Before beginning any research, the protocol has to be approved by the ethics committee. This protocol has been reviewed and approved by the Xin-Hua Hospital Institutional Review Boards (IRBs) and COMIRB, including:

- Nutrition monitoring design;
- Participants recruitment, enrollment and interventions;
- Daily interviews, biological measures and specimen collections;
- Data analysis and publication of findings.

Senior staffs of this project have been certified in “Ethics in Research” by the Office of Human Protection in Research. Field personnel will be trained in the most important aspects of ethics in research by senior staff.

2. Principles of human research ethics

A. Respect for persons

- The autonomy, self-determination.
- Protection of vulnerable groups (those with limited education, the poor, those with difficult access to heath services, women).
- The informed consent:
  - Respect for persons is embodied in the Informed consent process.
  - Informed consent is designed to empower the individual to make a voluntary informed decision regarding participation in the research.
  - Potential research participants must fully comprehend all elements of the informed consent process.

B. Beneficence

- Physical, mental and social well-being. Risks reduced to a minimum.
  - Protection of the participant is the overriding responsibility of the researcher
  - Scientific correctness
  - Appropriate informed consent
  - Confidentiality protection
  - Conduct research according to protocol
  - Compliance with Ethics Committee (reporting adverse events, protocol violations and participant complaints)
  - Post-study: long term interests of participants

- Researcher's Human Qualities
    - Integrity
    - Respect
    - Compassion
    - Professionalism
    - Courtesy
    - Sensitivity

C. Justice

- Distribution of risk and benefit
- Equitable recruitment of research participants
- Special protection for vulnerable groups

3. Confidentiality

A. All information obtained from participants must be kept confidential. Data about participant cannot be associated with her/him as an individual.

B. Monitoring staff must maintain confidentiality of all information gathered from or about a participant:

- Information collected on the data forms;
- Contact information;
- Medical record abstractions.

C. Do not discuss a participant with anyone except other project staff:

- Information about a participant could be repeated to someone who could identify her/him;
- Monitoring result and other data collection materials like official medical records.

4. Safeguards to ensure confidentiality and protection of data

A. Materials with identifying information kept in a secure place.

B. Evaluation interviews conducted in privacy and at a convenient time for participant.

C. Participant's name can not connected to results when the data have been analyzed. The participant's ID number:

- Created by data management system;
- Identifies a study participant;
- On all forms and data files to replace name;
- Guarantees anonymity while allowing tracking of activities.

D. Data presented in aggregate

5. Components of informed consent

A. Research description

- Description of the nature of study, the objectives, expected responsibilities, procedures involved, study duration and explanation of randomization or placebo.

B. Description of risks

- Anticipated or foreseeable risks, physical, social and psychological risks, and culturally appropriate.

C. Description of benefits

- Reasonably expected, no exaggeration of benefits once research is ended.

D. Description of available alternatives

- Alternative procedures or treatment, advantages and disadvantages and availability.

E. Description of confidentiality

- Indicate persons or organizations who may have access to the information.

F. Compensation

- It is permissible to compensate participants for their time, travel and inconvenience; however this should not be so high as to unduly influence a potential participant's decision in the study.

G. Contacts

- Contact for research-related questions.
- Contact for concerns about rights as a participant, realistic and viable.
- Voluntary Participation, absolutely voluntary, right to discontinue at any time and no penalty for refusal.

6. Documentation of informed consent

- A key component to the process of informed consent is the signing or documentation of the consent form by the participant, the researcher and other individuals.

Section 3: Introduction to Study Documents

1. **Maintaining study documents**
   - The most recent version of the study protocol, consent form, manual of operations and data forms should be readily available for reference at the research unit in Shanghai monitoring team.
   - Supervisory staff should ensure that the correct version and date of study documents are being used.
   - If staffs have difficulty with completing forms or implementing study procedures, staffs should contact the core staff of Xi-Chou monitoring team, coordinator staff of Shanghai monitoring team, and Shanghai PI with their questions or feedback.
2. **Protocol**
   - The protocol provides the scientific basis for the study. The study objectives, rationale, study design, methodology, and statistical considerations of a clinical trial.
3. **Consent form**
   - The consent form describes the study procedures that participants are expected to take part in and adhere.
   - Compensation and contact information and other information regarding participation are included.
4. **Manual of operation**
   - The manual of operation describes in detail the procedures necessary to implement the study protocol.
   - The manual describes the study organization, preparations, recruitment and enrollment, baseline assessments, follow-up data collection, outcome ascertainment, and standard operating procedures.
5. **Data forms**

Data forms are used to acquire information (by interview, abstraction, or observation) that will be used to support the implementation of the trial and test the study hypotheses. A copy of each data form is included in the manual of operation.

Section 4: Study organization

Nutrition Monitoring Protocol Organization Chart

- Principal investigate of Shanghai: Sheng Xiao-yang
- Coordinator staff of Shanghai monitoring team: Xue Min-bo, Shen Li-xiao, Wang Jun-li, Sun Qian-qian, Zhou Li-li, Hu Yan-qi, Liu Jin-rong.
- Core staff of Xi-Chou monitoring team: Jiang Tian-jiang, Li Qiong
- Coordinator staff of Xi-Chou monitoring team: Ding Xiao-hua, Fang Ze-wei, Tao Hua-xing, Yang Xiao-qiao, Qi Min, Chen Fang, Wang Jin-min, Yang En-xian, Nong Yu-hua
- Xi-Chou monitoring assessment team: Li Qiong, Li Gui
- Shanghai laboratory assistant: Yu Xiao-gang, Liu Jin-rong
- Xi-Chou laboratory assistant: Liang Ru-chao
- Shanghai trained BSID specialist: Zhou Li-li, Hu Yan-qi, Shen Li-xiao
- Data entry staff: Sun Qian-qian, Wang Jun-li, Zhou Li-li, Hu Yan-qi, Liu Jin-rong
- Driver in Xi-Chou: Lu Nian-yong

Section 5: Identification, Recruitment and Enrollment of Participants

Identify potential participants (mother/child), and mobilization, recruitment and enrollment of the participants. Complete data form NIM 1 (Birth and Screening Log), NIM 2 (Screening Form), and NIM 3 (Enrollment Form).

1. Recording births

Complete data form NIM 1 (Birth and Screening Log), and identify potential participants.

- Available birth registry data will be obtained from the local health center or other available registry by coordinator staff of Xi-Chou monitoring team.
- NIM 1 (Birth and Screening Log) will be completed by core staff of Xi-Chou monitoring team. Coordinator staff of Shanghai monitoring team will provide ID number. ID number will be used in other data forms, for substituting the name of the participant.
- Organized by coordinator staff of Xi-Chou monitoring team, community doctors will identify and recruit potential participants, and contact to enroll once a month.
- NIM 1 (Birth and Screening Log) will be completed by core staff of Xi-Chou monitoring team and coordinator staff of Shanghai monitoring team together.

2. Screening of participants

Complete data form NIM 2 (Screening Form), according to NIM 1 (Birth and Screening Log).

- On the monthly meetings for screening and enrollment of participants, complete data form NIM 2 (Screening Form).
    - Inclusion criteria (all three required for inclusion):
        - The infant must be between 3 and 5.5 months of age at the time of enrollment.
        - The mother must be breastfeeding the child as a primary source of food. But, it is not required that the child be exclusively breastfed in order to be eligible for the study.
        - The mother should be intending to breastfeed the child to at least 12 months. Again, it is not required that the mother exclusively breastfeed the child through 12 months.
    - Exclusion criteria (if any one of these is present, infant is not eligible):
        - The mother feeds the infant formula daily or most days.

- The mother intends to feed her infant formula daily or most days.
- The infant has a birth defect.
- The infant is a twin, triplet or other high-order birth
- The infant has been diagnosed with a neurological defect or if the infant has experienced a seizure, such as poor (retarded) development, neural tube defect/ spina bifida/ hydrocephaly/ brain malformation, very high tone/ stiffness or very low tone/ floppiness/ severe weakness, seizures/ fits/ epilepsy.

3. Enrollment of participants

Complete data form NIM 3 (Enrollment Form), when the participant is eligible during the screening process.

- When monthly meetings for screening and enrollment of participants, complete data form NIM 2 (Screening Form), and if the participant is eligible, complete data form NIM 3 (Enrollment Form).

Section 6: Study Food Dispensing and Storage

Study food dispense and requirements for appropriate storage.

1. **Fresh lean pork**

- Pork will be provided by assigned supplier in local farm product market;
- Handi-wrap will be used to package pork, 50g per portion;
- According to daily requirements, coordinator staff of Xi-Chou monitoring team will supervise the purchase of lean pork and deliver to each community doctor daily or at least twice of week;
- Meat must be stored in refrigerator as soon as possible. The meat was been keep in cooler no more than one day, freeze no more than one week.
- Mother will bring her child to community doctors' home to eat it, or community doctors will distribute it to each participant through daily home visit and supervise mother feeding it to child.
- Remaining cooked meat can be fed again up to 2 hrs later provided it is covered and stored in cool place. If infant is only just starting to eat meat, mother may chose to save half of meat to cook and feed later in the day.
- Initially minced pork should be fed alone or with minimum of rice cereal. When infant is a little older and eats 2oz of pork/day, other foods can be added to the meal in progressively increasing quantities.

2. **Fortified cereal**

Regulations do not allow addition of a separate micronutrient mix to cereal grain flour, therefore:

- Nestle fortified cereal will be purchased by Shanghai monitoring team from the market.
- Fortified cereal will be packaged into 20g bag.
- According to daily requirements, core staff of Xi-Chou monitoring team will deliver fortified cereal to each community doctor weekly through coordinator staff of Xi-Chou monitoring team.
- Cereal must be stored at room temperature; room should also be well ventilated and protected from flood, leakage of water, rodents or other vermin.
- Mother will bring her child to community doctors' home to eat it, or community doctors will distribute it to each participant through daily home visit and supervise mother feeding it to child.
- Save small samples of fortified cereals for analysis at time of each purchase.

3. Ordinary cereal

- Product is produced by assigned store in local market and supervised by Xi-Chou monitoring team.
- The ordinary cereal is made of sticky rice and sugar, with the ratio of 2:1.
- The ordinary cereal will be packaged into 20g bag.
- According to next month requirements, core staff of Xi-Chou monitoring team will notify the store two weeks in advance. Cereal will be stored less than 2 months.
- According to daily requirements, core staff of Xi-Chou monitoring team will deliver it to each community doctor weekly through coordinator staff of Xi-Chou monitoring team.
- Cereal must stored at room temperature; room should also be well ventilated and protected from flood or leakage of water, rodents or other vermin.
- Mother will bring her child to community doctors' home to eat it, or community doctors will distribute it to each participant through daily home visit and supervise mother feeding it to child.
- Save small samples of ordinary cereals for analysis at time of each purchase.

Section 7: Assessment Visits

Assessment visits of nutrition monitoring will be performed at ages 6, 7, 9, 12, 15 and 18 months, including short diet records, sub-sample 24-hr diet records, and anthropometry. At 7 months, only short diet record required to determine meat consumption. Developmental testing performed only at ages 12 and 18 months. Blood sample only at 18 months (except for those in metabolic study); stool specimens at ages 6, 9, 12 and 18 months.

24-hr diet records, anthropometry, blood and stool specimen collections will be accomplished within a two week window (i.e. ± 1 week) around each age; Developmental testing will be accomplished within a four week window (i.e. ± 2 week) around each age.

1. Diet Records:

A Questionnaire

To be completed at ages 6, 7, 9, 12, 15 and 18 moths by assessment team by questioning primary caregiver. Fill in data form NIM 6, Section B. This part will be accomplished by coordinator staff of Shanghai monitoring team with assistance of Xi-Chou monitoring assessment team.

B 24-hr diet records

24-hr dietary recalls will be obtained at ages 6, 9, 12 and 18 months during the assessment visits. These records to be collected on a convenience sample of 100 participants in each group, with repeat data collections in 30 participants per group in order to determine intra-individual variation.

2. Anthropometry

Anthropometry will be obtained at 6, 9, 12, 15 and 18 mo during the assessment visits, and then complete data form NIM 6, Section C. This part will be accomplished by Xi-Chou monitoring assessment team.

A. Measuring weight

1. Remove the infant's clothing to a dry diaper.
2. Place the child on his/her back or sitting on the tray of the scale. The child must be centered in the tray and is not touching anything off of the scale tray including other parts of the scale.
3. Read and call out the measurement to the nearest 10 g.
4. Record the weight on Sheet 6, Section C. Make sure it is accurate and legible.
5. Remove the child from the tray of the scale and zero it in preparation for the

next measurement.

6. Two measurements must be taken and recorded on Sheet 6.
7. If the difference between first two measurements >10g, a third measure must be taken and recorded.

B. Measuring head circumference

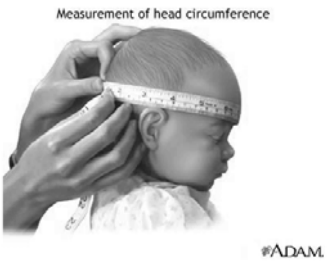

1. Use graduated tape measure for all measurements.
2. Child should be seated or decubitus position.
3. The tape is positioned just above the eyebrows and placed posteriorly over the occiput to give the maximum circumference. Pull the tape just tight enough to compress hair. Please see Picture 1 for correct positioning of the tape.
4. Head circumference is recorded to the nearest 0.1 cm.
5. Record the numerical value immediately on Sheet 6.
6. Two measurements must be taken and recorded on Sheet 6.
7. If the difference between first two measurements >0.2cm, then a third measure must be taken and recorded.

C. Measuring recumbent length

1. A team of two Measurer and Assistant will work together to measure recumbent length.

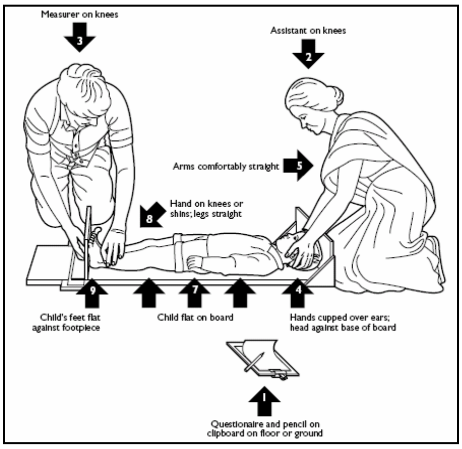

2. The Assistant must remove hats, barrettes, shoes and socks.
3. The Measurer places the sliding foot piece at the end of the measuring board and checks to see that it is sliding freely.
4. The Assistant has the child lay down on his/her back on the measuring board and positions himself/herself directly behind the child's head.
5. The Measurer positions himself/herself on the right side of the child to hold the foot piece with the right hand. Note: While the infant is on the measuring board, hold and control the child so that he/she will not roll off or hit his/her head on the board.
6. The Measurer holds the child securely at the waist while the Assistant positions the head.

7. The Assistant cups her hands over the child's ears and places the child's head against the headpiece.
8. If the head is not against the headpiece, the Measurer should hold the child at the waist and lift or slide the child towards the headpiece.
9. The Assistant should hold the child's head at all times and guide the head into position.

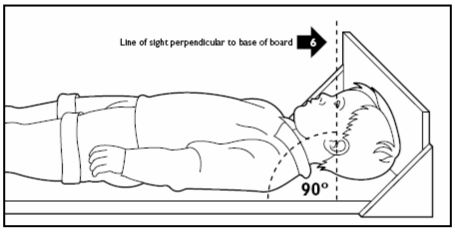

10. The Assistant should check to be sure that the child's head is in the correct position. The line from the hole in the ear to the bottom of the eye socket (Frankfort Plane) should be perpendicular to the board or table.
11. The Measurer and Assistant should ensure to position the child's body so that the shoulders, back and buttocks are flat along the center of the board.
12. The Measurer should place hands on both knees or shin to assure legs are straight and fully extended. Feet must be perpendicular to the board and the full surface of feet must be flat against the foot piece.
13. When the child's position is correct, the Measurer reads and calls out the length measurement to the nearest tenth of a centimeter. Record immediately the measurement on Sheet 6, Section C. Check to make sure it is accurate and legible.
14. Two measurements must be taken and recorded on Sheet 6.
15. If the two measurements do not agree within 0.4cm, a third measurement must be taken.

3. Developmental testing

The Bayley Scales of Infant Development, 3nd Edition**,** (BSID III) will be used to assess the infant's development at 12 and 18 months of age.

All participants will accept the test by appropriately trained and experienced testers with assistance of Xi-Chou monitoring assessment team.

This test should be accomplished within a four week window (i.e. ± 2 week) for each participant around each age.

4. Blood and stool specimen collections

Blood specimen should be collected at 18 months of age, total 5ml.

This part will be completed by Shanghai & Xi-Chou Laboratory assistant with assistance of Xi-Chou Monitoring Assessment research worker.

A small stool sample will be collected at 6, 9, 12 and 18 month. This sample can be stored at room temp for 6 days.

Section 8: Home Visits by Community Doctor

Home visits by community doctor including: potential participants' home visits before 5-6 m at least once; participants' weekly home visits for monitoring, food preparation & feeding, morbidity monitoring, and nutrition education messages (rationale and delivery). For the meat group, the community doctor is responsible for daily delivery of uncooked meat if mother is unable to collect from doctor's home..

1. **Potential participants home visits (Interim Home Visit)**
   - At least one interim visit will be made between enrollment (age 3-5.5months) and before the infant reaches 6 months of age.
   - Purpose of the visit:
     - Remind the mother of the Nutrition Monitoring and assessment visits;
     - Encourage continued breastfeeding, including exclusive breastfeeding as much as possible
     - Encourage responsive feeding;
     - Encourage mother to plan to start complementary foods when the infant is 6 months old

A. Delivery of study foods
   - Coordinator staff of Xi-Chou monitoring team will distribute meat, fortified cereal or ordinary cereal to community doctors; Meat will be distributed at least once a week.
   - The community doctors will be responsible for study food dispensing to each participant daily, or provide to the mother if she comes to the doctor's home. The doctor may also choose to feed the participants at his/ her home.
   - Cereal can be delivered to participants' homes weekly by the community doctors
   - The community doctors will be in charge of monitoring the feeding process weekly.
   - Complete data form NIM 4, and submit it to Coordinator staff of Xi-Chou monitoring team weekly.

B. Study food preparation & feeding
   - When the study food is prepared by community doctors or mothers, please notice:
     - Wash your hands with clean soap and water before preparing food and feeding.

  - Wash baby's hands with soap and water before feeding
  - Use clean water for preparing food
  - Feed the baby with clean spoon, cup or bowl, and never use a feeding bottle
  - Give the food soon after preparing
- Lean pork
  - One 50g packet of lean pork should be fed to the child each day;
  - Lean pork can be steamed, boiled, or stewed, and a paste or puree is made, then be provided to the infant straight;
  - The lean pork should be fed as a single food to begin with;
  - After the infant has learned to eat it well, the infant can eat other foods as ages;
  - After the infant has learned to eat it well, it may be added to a small amount of other foods.
- Fortified cereal
  - Boiling water, and keep 5 minutes longer;
  - Put the boiling water into the plastic box, and add the full 20 g packet of fortified cereal;
  - Stir until it thickens and a purée consistency is achieved;
  - Cool and feed to child (mothers should be encouraged to test the cereal before feeding it to the child);
  - The fortified cereal should be fed as a single food to begin with;
  - After the infant has learned to eat it well, the infant can eat other foods as ages;
- Ordinary cereal
  - Boiling water, and keep 5 minutes longer;
  - Put the boiling water into the plastic box, and add the full 20 g packet of ordinary cereal;
  - Stir until it thickens and a purée consistency is achieved;
  - Cool and feed to child (mothers should be encouraged to test the cereal before feeding it to the child);
  - The ordinary cereal should be fed as a single food to begin with;
  - After the infant has learned to eat it well, the infant can eat other foods as ages;
- Feeding the study foods

- For young infants, the lean pork, fortified or ordinary cereal should be the only food (in addition to breast milk) the infant eats until he or she can eat it all.
- At the beginning (around 6 month of age), mothers should encourage, but not force, the child to eat the entire packet each day.
- At the beginning, the young infants eat only a small proportion at one feed, the mother may split the daily ration in two to three feeds to assure that the entire packet is eaten during the day.
- If the daily cooked (or moistened) portion is split into 2 feedings, the unused portion should be stored in the study container until use. The food should be used within ~ 2 hr, or be heated to feed again, never feed food which is overnight to child.
- For the older infant (e.g. after 7 months), if the study is added to another food item(s), instruct the mother to first feed the study food.
- For 12-18 month olds, encourage self-feeding (e.g. picking up meat cubes, using spoon for cereal or purees). Guide the mother help her child eat the entire packet, and avoid spatter and waste.

C. Monitoring Morbidity

Awareness and report of study food eating and infectious disease morbidity will be greatly facilitated by the weekly home visits from 6 to 18 mo of age. Fill in data form NIM 4 /5.

- Monitoring respiratory illness, diarrhea and any febrile or severe illness
    - the community doctors should conduct the weekly home visits with participants, and record on data form NIM 4 faithfully.
    - For each participant, community doctor should complete a data form NIM 4 weekly, and submit it to coordinator staff of Xi-Chou monitoring team, finally gather to core staff of Xi-Chou monitoring team;
    - If community doctor observes the child is ill during the home visit, fill it on data form NIM 5;
    - During child's illness, community doctor should complete a data form NIM 5 every day, until the child is recover. Submit it to coordinator staff of Xi-Chou monitoring team, finally gather to core staff of Xi-Chou monitoring team.
    - If symptoms of severe illness are reported by the mother/ caregiver or if

the community doctor observes symptoms indicating severe illness, the community doctor must refer the child to the nearest hospital for immediate care.

- Requirement of Adverse Event Report
  - If the child is hospitalized for any reason (disease or trauma), the community doctor should inform coordinator staff of Xi-Chou monitoring team immediately, and report to core staff of Xi-Chou monitoring team.
  - Completion of form NIM 10 (Adverse Event Report) by core staff of Xi-Chou monitoring team.
  - The community doctor should follow up the child in hospital, and to obtain the final diagnosis, treatment and duration of hospitalization

D. Dissemination of Educational messages

Nutrition education messages for Breastfeeding and Complementary Feeding will be disseminated during the weekly home visits and interim home visit by the staff of Xi-Chou monitoring team and community doctors. (See detailed information in appendix 1)

- Nutrition education messages for Breastfeeding
  - Breast milk is an important source of nutrition for your baby
  - Continue breastfeeding, even after starting solid foods
  - Continue breastfeeding until child is two years old
- Nutrition education messages for Complementary Feeding
  - Give your baby thick, not thin, foods
  - Give your baby at least three meals a day, every day
  - Give your baby many different foods

Be wise in how frequently you repeat the same advice depending on your assessment of each household and the apparent nutritional status of the child. Give time to respond to questions.

Section 9: Administration of Data Forms

Monitoring staff should complete data forms properly, following the "Question – by – Question Specifications". Make corrections to a data form in accordance with the regulation when make a mistake. Input data and submit to Shanghai monitoring team in time.

1. General instructions for completion of data forms

- Complete data forms and check to make sure they are accurate and legible. The detail can be seen in the Sheet Specifications. (appendix 2)

2. Making corrections to completed data forms

- To make a correction to a data form, cross out the incorrect data with one line (using a black ink pen) and write the correct data outside the data field in black ink.
- The staff person making the correction should initial and date the correction
- The use of correcting fluid or "white out" is prohibited.

3. Submitting completed data forms

- Data form for which community doctor is responsible (combination of NIM 4 and NIM 5), should be submitted to their coordinator staff of Xi-Chou monitoring team for review on a weekly basis.
- After a preliminary verification, coordinator staffs of Xi-Chou monitoring team gather the weekly data forms from the community doctors, and submit to core staff of Xi-Chou monitoring team.
- Finally the data forms completed by community doctors and staff of Xi-Chou monitoring team should be submitted to data entry staff.

4. Data entry

- Data entry staff should archive completed data forms in time, and data sheets should be stored in a locked filing cabinet;
- Data entry staff should submit the data to Shanghai monitoring team weekly.
- Coordinator staff of Shanghai monitoring team will be responsible for receiving the data and recovery them to English version of the forms, except NIM 4.
- At the same time of recovery, verify the accuracy of the data.

Module 12: Study Management

1. The PI will go to Xi-Chou to supervise and direct the implementation of the monitoring once a month.

2. The core staff of Xi-Chou monitoring team will meet with coordinator staff of Xi-Chou monitoring team at least once a week.

- Deliver the completed data forms to the core staff of Xi-Chou monitoring team;
- Discuss problems encountered in implementing monitoring procedures;
- Identify and provide solutions;
- Plan activities for the forthcoming weeks: home visits, assessments and delivery of study food.

Appendix 1: Educational messages

6-12 month old infants

Feed thickened gruels (**"Give your baby thick, not thin, foods."**):

- Babies and young children have a high need for energy (calories) because they are growing and they get more and more active.
- After 6 months, breast milk alone is not enough to provide enough energy and nutrition. The "gap" between the child's needs and what he or she can get from breast milk gets bigger. If this gap is not filled, the baby will stop growing or grow slower (even if the baby looks healthy or does not ask for more food).
- Thin foods have more water and less nutrition, and do not help baby grow, because they do not provide energy or nutrients (protein, vitamins).
- Making the foods with less water makes them more nutritious
- A thick puree should be too thick to drink, but thin enough to be eaten with a spoon.
- Adding fatty or oily foods (butter, oils, ghee, or ground nut paste) makes the thick porridge softer and easier to eat.
- If the family makes soups, then solid pieces can be taken from this and mashed to a puree (and a little oil added to make this soft and higher in energy).

Feed infant/toddler at least 3 times a day; (**"Give your baby at least 3 meals a day, every day"**):

- The energy (and food) needed by the child increases as the child gets older, bigger and more active.
- Babies have small stomachs; they need to be offered foods several times each day to help them get enough to grow.
- Babies and children can't (or don't) always tell you that they are hungry but they will eat if offered food.
- Keep breastfeeding and also give the baby meals with solid foods 3 times a day.
- In 6-month olds, start with 1 or 2 small spoons of food 2 times a day, but soon get to 3 times/day).
- By 9 months of age, a child can eat several spoonfuls (~ ½ cup) at each meal.
- Find 3 times during the day mother or care provider can sit with the baby to

offer foods by spoon. This might be in the morning, in the middle of the day, and in the evening or afternoon.

Maximize local food diversity (**"Give your baby many different foods"**)

- No single food provides everything the baby needs.
- Other foods must be eaten with the "staple" (maize, rice, roots, etc) to fill the energy and nutrient gaps.
- By 6 months babies are able to digest all foods.
- Babies may be surprised by new tastes and textures; this does not mean they do not like the food.
- They need practice each day to become familiar with the food.
- Types of foods that are best to fill the gaps:
  - Pulses (beans, peas, groundnuts) and oil seeds (sesame seeds)
  - Foods from animals (eggs, cheese, milk)
  - Dark-green leaves and orange-colored fruits and vegetables
  - Oils and fats

<u>12-18 months old children</u>

Continue to reinforce same messages, but recognize that as the infant grows and develops, the specific implementation of the messages will change.

- As the baby gets older, increase the amount and the variety of foods (as possible); an older baby needs to eat more to meet his/her nutrition needs.
- The child should be allowed to hold the spoon and begin to feed him/herself. At 12 months, the mother will still need to assist some, but the child should be encouraged to practice using the spoon. The child will also enjoy picking up pieces of food (e.g. the meat cubes, or for the cereal group, other soft foods, such as fruits or cooked vegetables)
- Family foods can be mashed to make thick puree/mixture;
- Continue to offer at least 3 meals per day in addition to breastfeeding as the child wants.
- Give the child many different foods.
